# Digital press shops: Data of an online survey among press shop experts

**DOI:** 10.1016/j.dib.2021.106880

**Published:** 2021-02-13

**Authors:** Marius Groß, Kolja Lichtenthäler, Georg Bergweiler, Peter Burggräf

**Affiliations:** a Laboratory for Machine Tools and Production Engineering (WZL), RWTH Aachen University, Aachen, Germany; b Department of Mechanical Engineering, Chair of International Production Engineering and Management (IPEM), University of Siegen, Siegen, Germany

**Keywords:** Press shop, Body in white, Part tracking, Digitalisation, Process connection, Automotive, Process data, Data mining quality information

## Abstract

Modern automotive press shops are reaching their process limits due to increasing demands on car body shapes. At the same time, transmission of information and readjustment in the event of quality losses because of process errors is still largely controlled manually. The survey presented here, deals with better connected processes as well as data acquisition, and track and trace applications in press shops. The survey was directed to experts from the automotive industry and is to determine how automated and connected the processes in press shops already are. The survey was conducted from March till April 2020. With a total of 24 questions, an attempt is made to gain a comprehensive picture of the current status and the existing potential regarding smart press shops. In addition to questions on the marking and tracking of pressed parts, the objective is to find out which process data is already being recorded today and what conclusions can be drawn from it regarding the expected part quality. The evaluation of the survey is intended to build the basis for research activities on smart, connected press shops.

## Specifications Table

**Table:** 

Subject	Industrial and Manufacturing Engineering
Specific subject area	Press shops of the automobile industry, Data and information about connectivity in modern press shops
Type of data	Table Chart
How data were acquired	Data was gathered using an online survey and converted into excel format.
Data format	Data is in raw format and has been processed in charts. The raw data is provided in excel format.
Parameters for data collection	The target group of the survey was experts of the automotive manufacturing industry. Experts were contacted through the network of the Laboratory for Machine Tools and Production Engineering (WZL), RWTH Aachen University.
Description of data collection	The data was conducted through an online questionnaire, which was delivered to various experts of the German automotive industry. The questionnaire was in German.
Data source location	Information was collected from Laboratory for Machine Tools and Production Engineering (WZL), RWTH Aachen University in Germany.
Data accessibility	Data is supplied with the paper.

## Value of the Data

• The data gives insights to the current status of press shops in automotive industry and an overview of specific process characteristics of press shops. It also provides information on the potentials and restriction of using track & trace systems in press shops to track body parts amongst the production chain.
• The data will be useful for production scientist, press shop planers, and quality planers. Researchers can use the data to identify further research topics and studies. The data can be used to get insight of the characteristics of production systems in automotive press shops.
• The data can be used as input information to develop and implement a real demonstrator of a potential track and trace system in the production of body parts. It can provide an information basis to identify specific areas of the production where the need of a track and trace system of body parts is the highest.

## 1 Data Description

The data set provides an insightful information based on survey data on knowledge and practice among manufacturing automation and data networking in automotive press shops. The data include three parts of information. The first part shows the company and participant information, which are shown in Fig. 1, Fig. 2, Fig. 3, Fig. 4. Fig. 5, Fig. 6, Fig. 7, Fig. 8, Fig. 9, Fig. 10, Fig. 11, Fig. 12, Fig. 13 shows the answers of the participants regarding the current status of the production in automotive press shops. The last part of the data set provides information about the data and information flows in the press shop (Fig. 14, Fig. 15, Fig. 16, Fig. 17, Fig. 18, Fig. 19, Fig. 20, Fig. 21, Fig. 22, Fig. 23, Fig. 24).

**Fig. 1:**
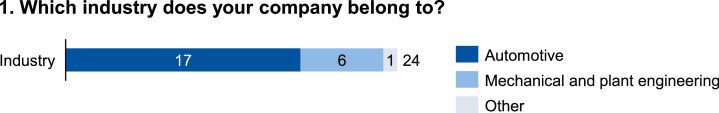
Industry of the participants.

**Fig. 2:**
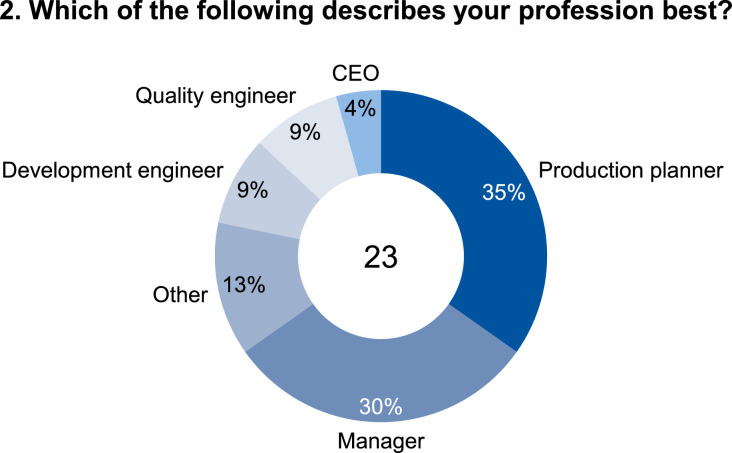
Profession of the participants.

**Fig. 3:**
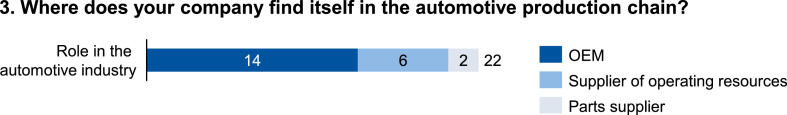
Role of the company of the participants.

**Fig. 4:**
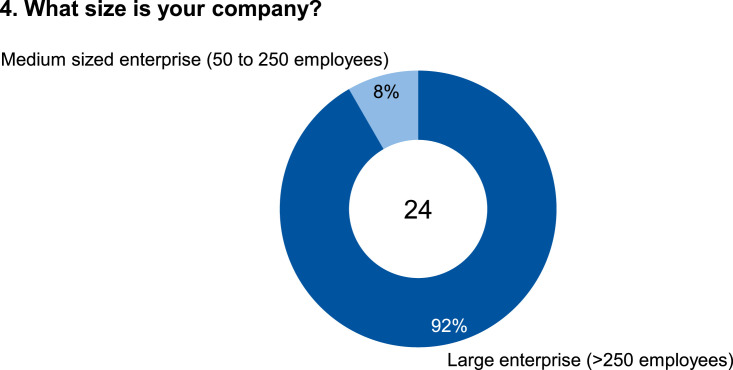
Size of the company of the participants.

**Fig. 5:**
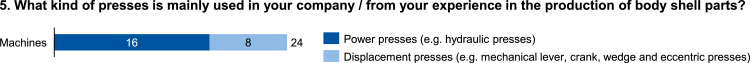
Types of presses in the press shops.

**Fig. 6:**
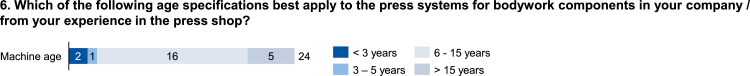
Machine age of the presses in the press shop.

**Fig. 7:**
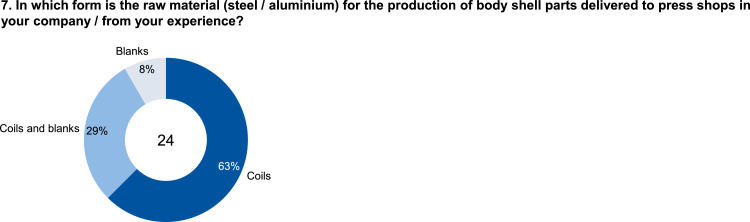
Form of delivery of the raw material.

**Fig. 8:**
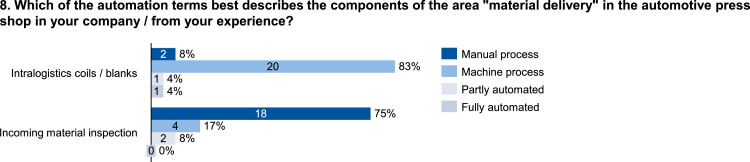
Form of automation in the material delivery area.

**Fig. 9:**
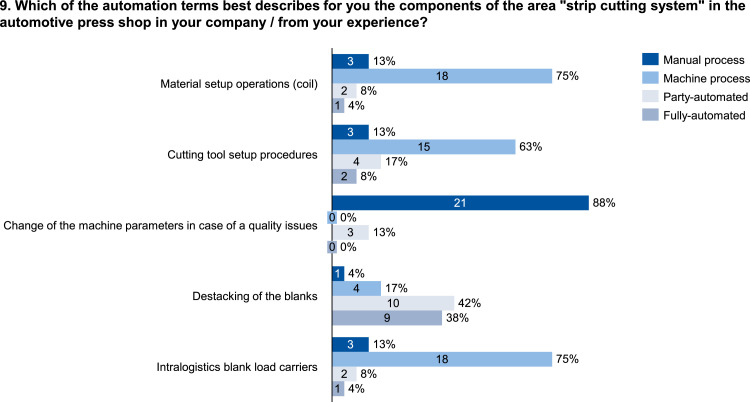
Form of automation in the strip cutting system.

**Fig. 10:**
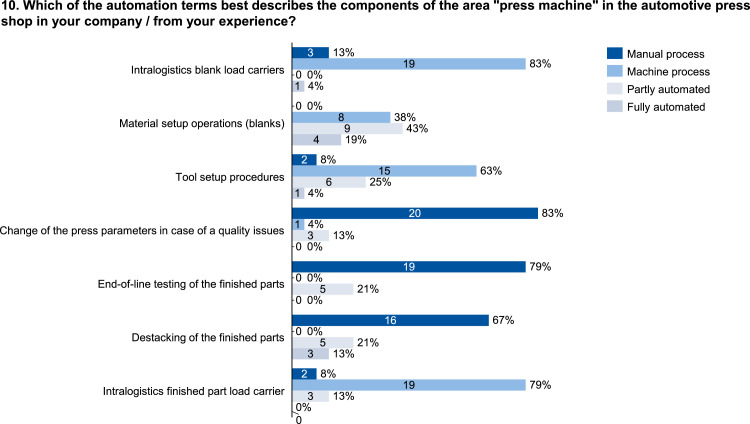
Form of automation in the press machine.

**Fig. 11:**
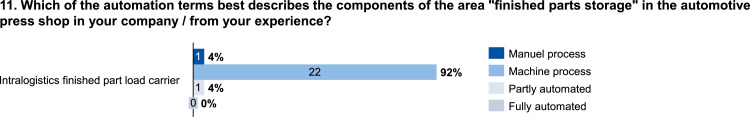
Form of automation of the finished parts storage.

**Fig. 12:**
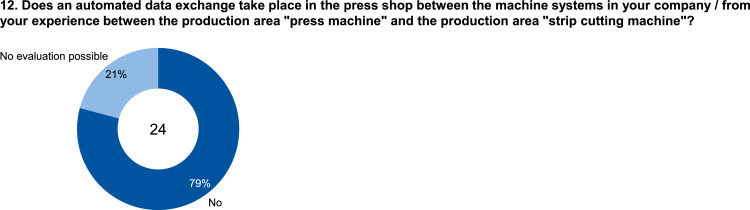
Process of data exchange between the press machine and cutting machine.

**Fig. 13:**
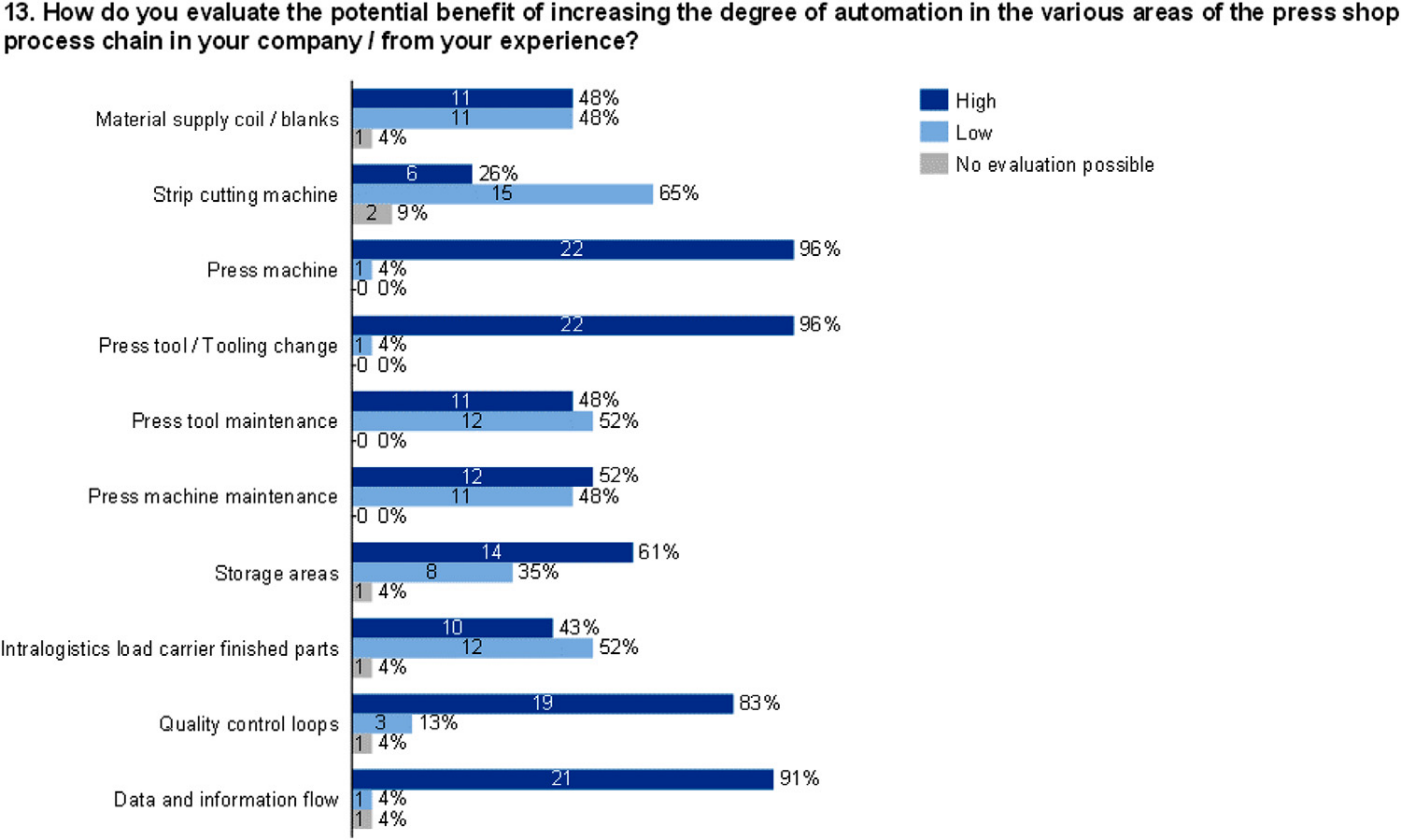
Evaluation of the potential benefit of an increase of automation in the press shop.

**Fig. 14:**
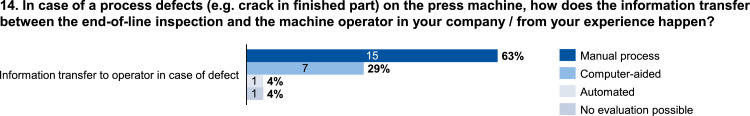
Process of information transfer between end-of-line inspection and machine operator in case of a defect.

**Fig. 15:**
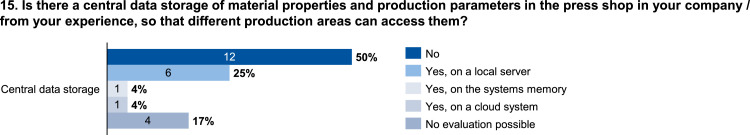
Accessibility of data in different production areas.

**Fig. 16:**
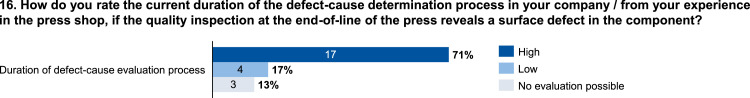
Duration of defect-cause evaluation process.

**Fig. 17:**
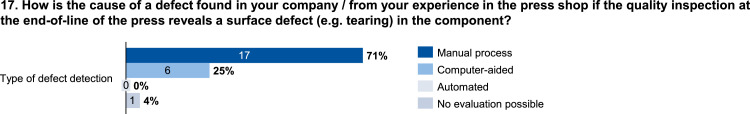
Type of defect detection in the end-of-line area.

**Fig. 18:**
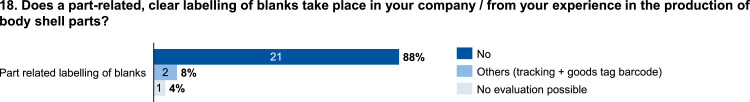
Usage of part related labelling of blanks.

**Fig. 19:**
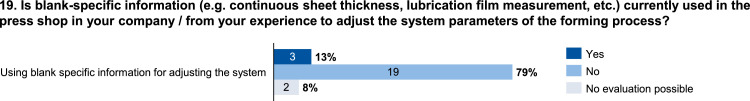
Usage of blank-specific information to adjust the manufacturing system parameters.

**Fig. 20:**
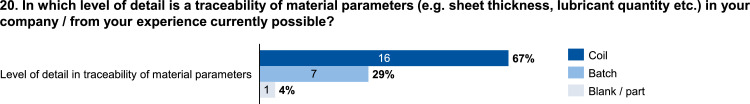
Traceability level of detail of material parameters.

**Fig. 21:**
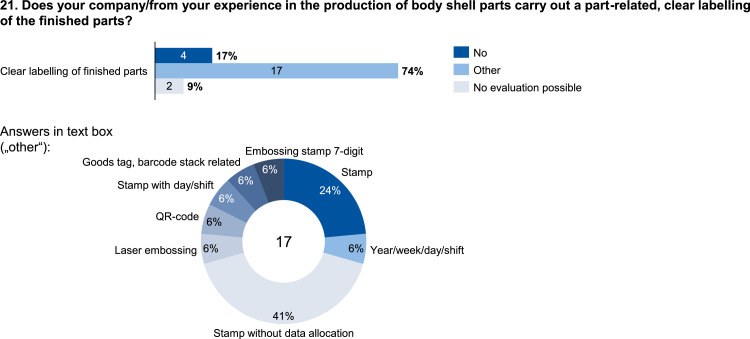
Usage of part-related labelling of finished parts.

**Fig. 22:**
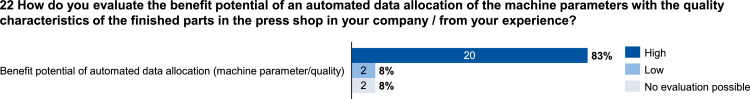
Benefit potential of automated data allocation of machine parameter and quality characteristics of finished parts.

**Fig. 23:**
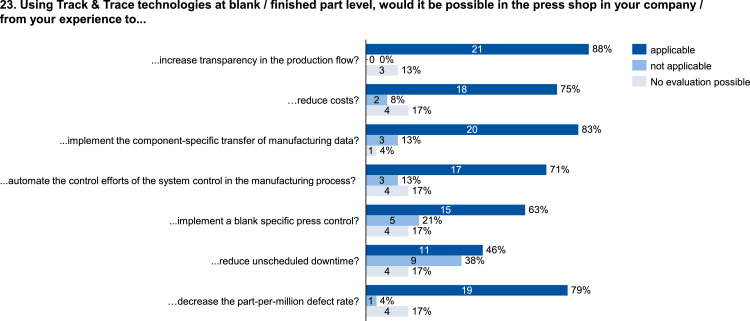
Evaluation of the potential benefits of using track & trace technology at blank and finished part level.

**Fig. 24:**
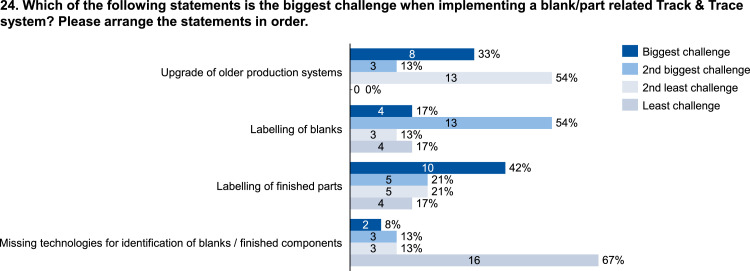
Evaluation of the challenges of implementing a blank or part related track & trace system.

## 2 Experimental Design, Materials and Methods

To collect empirical data on the characteristics of the production processes and to identify deficits in data acquisition and transmission of information in the press shop, a survey is carried out. The survey should provide an overview of the current characteristics of the production processes in automotive press shops and is performed by using an online questionnaire. The collection of data in a survey is a frequently used method aiming to falsify or confirm hypotheses in empirical research [1]. The instrument used in this method is a scientific questionnaire, answered independently by the respondents. The methodology used in this paper is described using the criteria shown in Table 1. The development of the questions for the questionnaire took place in the course of workshops and interviews with production engineering scientists and industry representatives, especially from the automotive industry and factory planning.

Since the survey pursues the goal of obtaining an up-to-date picture of the data collection and the transmission of information in press shops, a quantitative questionnaire is used. A fully standardised questionnaire with closed questions allows the respondents to select multiple-choice answers that they consider to be appropriate. Within the framework of this study, the survey is designed to question individuals. The questionnaire is implemented as an electronic version, which is send out by email.

The questionnaire is addressed to experts. In this context, experts are defined as persons who have access to relevant knowledge, decision-making processes, or groups of persons [2]. In terms to this paper, the status of an expert is therefore based on expertise in the area of the press shop in the automotive industry, as well as on experience in forming technology and data networking. Particular attention is given to the fact that the respondents are either experts in the field of forming technology or occupy a position in a subject area covered by the survey. This is verified in the survey by answering introductory questions on the background of the person completing the form, whereby no personal information is requested. The survey was distributed to the experts through various newsletters of the Laboratory for Machine Tools and Production Engineering of RWTH Aachen. By using the network of the department leader of forming processes a broad number of experts could be reached. Overall, 24 respondents took part in the survey, however not all of them answered all questions. Furthermore, the answers of the participants do not represent the whole industry branch, therefore the survey does not claim to be representative. Nevertheless, it gives an indication of the state of digitisation in automotive press shops. As shown in the Figs. 1 and 2, most respondents work for car manufactures in Germany. Considering the fact, that there are only a few press shops experts in the automotive industry, the indication be useful to motivate more research and development in the digitisation of press shops as mentioned in the section “value of the data”.

**Table 1:** Survey criteria.

**Paradigm of the survey**	**Quantitative**		Qualitative	
**Degree of structure**	Non-standardised	Semi-standardised	**Fully standardised**	
**Mode of the survey**	Paper pencil Questionnaire		**Electronic Questionnaire**	
**Dissemination of the survey**	Postal	**Online**	Mobile	Sampling
**Type of respondents**	Involved people		**Experts**	
**Scope of the survey**	**Single person**		Group	

## Ethics Statement

In the authors' understanding, the work complies with the ethical requirements for publication in *Data in Brief*. All participants were informed that the survey results will be published. Thereby they gave their consent to participate in the survey and agreed to the publication.

## CRediT Author Statement

**Marius Groß:** Methodology Investigation; Writing - Original Draft; Writing - Review & Editing; **Kolja Lichtenthäler:** Methodology Investigation; Writing - Original Draft; Writing - Review &Editing; **Georg Bergweiler:** Writing - Review & Editing; **Peter Burggräf:** Supervision.

## Declaration of Competing Interest

The authors declare that they have no known competing financial interests or personal relationships, which have, or could be perceived to have, influenced the work reported in this article.

## Appendix Supplementary materials

Image, application 1

Image, application 2

Image, application 3

Image, application 4
