# Supplementary material for: Digital press shops: Data of an online survey among press shop experts
Source: Data Brief. 2021 Feb 13;35:106880. doi: 10.1016/j.dib.2021.106880 (PMC7907777; doi:10.1016/j.dib.2021.106880)
Supplement: Supplementary file 3 [file mmc3.pdf]

Manufacturing automation and data networking in the automotive press shop

Company and participant information

Digital technology offers new possibilities for the intelligent production of sheet metal components. Especially in the field of production automation, new efficiency potentials can be raised by networked production processes.

The aim of this survey is,
- to characterize the actual state of the individual production areas of the automotive press shop based on their process automation,
- to describe the data / information flow of material properties and manufacturing parameters in the press shop
- to identify the key points for implementing an automated closed information chain in the press shop.

Which industry does your company belong to?

- ◯ Automotive
- ◯ Mechanical and plant engineering
- ◯ Software and IT
- ◯ Logistics
- ◯ Other ________

Which of the following describes your profession best?

- ◯ CEO
- ◯ Manager
- ◯ Production planner
- ◯ Development engineer
- ◯ Quality engineer
- ◯ Worker
- ◯ Other ________

Where does your company find itself in the automotive production chain?

- ◯ OEM
- ◯ Part supplier
- ◯ Supplier of operating resources
- ◯ Other [ ]

What size is your company?

- ◯ Micro enterprise (up to 10 employees)
- ◯ Small enterprise (10 to 50 employees)
- ◯ Medium sized enterprise (50 to 250 employees)
- ◯ Large enterprise (>250 employees)

Press shop - Production

What kind of presses is mainly used in your company / from your experience in the production of body shell parts?

- ◯ Displacement presses (e.g. mechanical lever, crank, wedge and eccentric presses)
- ◯ Power presses (e.g. hydraulic presses)
- ◯ No evaluation possible

Which of the following age specifications best apply to the press systems for bodywork components in your company / from your experience in the press shop?

- ◯ < 3 years
- ◯ 3 - 5 years
- ◯ 6 - 15 years
- ◯ > 15 years

In which form is the raw material (steel / aluminium) for the production of body shell parts delivered to press shops in your company / from your experience?

- ◯ Coils
- ◯ Blanks
- ◯ Coils and blanks
- ◯ No evaluation possible

The following questions serve to identify the automation characteristics in the different production areas in press plants. For this purpose 4 levels of automation were defined.

Manual process - manual implementation of the activity by a worker (e.g. manual destacking of components)

Machine support - The worker is supported in his work by a machine. The machine takes over the working movement, but the worker is still responsible for control and guidance. (e.g. manual control of the overhead crane for tool change)

Partial automation - Electronic controls take over both the working movement and the control of the production machines. (e.g. manual press tool selection, hall crane moves automatically to the tool position, manual coupling / uncoupling of the press tool, automated positioning at the destination)

Full automation - The automation technology takes over the task of controlling and monitoring the technical processes completely. (e.g. manual press tool selection, hall crane moves automatically to the tool position, automated coupling and uncoupling of the press tool, automated positioning at the destination)

Which of the automation terms best describes the components of the area "material delivery" in the automotive press shop in your company / from your experience?

The area "material delivery" includes the delivery of materials to incoming goods, incoming goods inspection, intermediate storage, and intralogistics for material provision to the next production area.

	Manual process	Machine Process	Partly automated	Fully automated
Intralogistics coils / blanks	◯	◯	◯	◯
Incoming material inspection	◯	◯	◯	◯

Which of the automation terms best describes for you the components of the area "strip cutting system" in the automotive press shop in your company / from your experience?

The area "strip cutting system" extends from the material loading of the system to the intralogistics of the load carriers for intermediate storage or to the next production area.

	Manual process	Machine process	Parly automated	Fully automated
Material setup operations (coil)	◯	◯	◯	◯
Cutting tool setup procedures	◯	◯	◯	◯
Change of the machine parameters in case of a quality issues	◯	◯	◯	◯
Destacking of the blanks	◯	◯	◯	◯
Intralogistics blank load carriers	◯	◯	◯	◯

Which of the automation terms best describes the components of the area "press machine" in the automotive press shop in your company / from your experience?

The area "press plant" extends from the material loading of the plant to the intralogistics of the load carriers to the finished parts warehouse.

	Manual process	Machine process	Partly automated	Fully automated
Intralogistics blank load carriers	◯	◯	◯	◯
Material setup operations (blanks)	◯	◯	◯	◯
Tool setup procedures	◯	◯	◯	◯
Change of the press parameters in case of a quality issues	◯	◯	◯	◯
End-of-line testing of the finished parts	◯	◯	◯	◯
Destacking of the finished parts	◯	◯	◯	◯
Intralogistics finished part load carrier	◯	◯	◯	◯

Which of the automation terms best describes the components of the area "finished parts storage" in the automotive press shop in your company / from your experience?

The area "finished parts warehouse" comprises the intralogistics of the finished parts load carriers as well as the handling of outgoing goods.

	Manual process	Machine process	Partly automated	Fully automated
Intralogistics finished part load carrier	◯	◯	◯	◯

Does an automated data exchange take place in the press shop between the machine systems in your company / from your experience between the production area "press machine" and the production area "strip cutting machine"?

◯ No evaluation possible

◯ No

◯ Yes

How do you evaluate the potential benefit of increasing the degree of automation in the various areas of the press shop process chain in your company / from your experience?

	Low	High	No evaluation possible
Material supply coil / blanks	◯	◯	◯
Strip cutting machine	◯	◯	◯
Press machine	◯	◯	◯
Press tool / Tooling change	◯	◯	◯
Press tool maintenance	◯	◯	◯
Press machine maintenance	◯	◯	◯
Storage areas	◯	◯	◯
Intralogistics load carrier finished parts	◯	◯	◯
Quality control loops	◯	◯	◯
Data and information flow	◯	◯	◯

Press shop - Data and information flows in the press shop

In case of a process defects (e.g. crack in finished part) on the press machine, how does the information transfer between the end-of-line inspection and the machine operator in your company / from your experience happen?

	Manuel process	Computer-aided	Automated	No evaluation possible
Information transfer to operator in case of defect	○	○	○	○

Is there a central data storage of material properties and production parameters in the press shop in your company / from your experience, so that different production areas can access them?

- ○ No evaluation possible
- ○ No
- ○ Yes, on the systems memory
- ○ Yes, on a local server
- ○ Yes, on a cloud system

How do you rate the current duration of the defect-cause determination process in your company / from your experience in the press shop, if the quality inspection at the end-of-line of the press reveals a surface defect in the component?

	Low	High	No evaluation possible
Duration of defect-cause evaluation process	○	○	○

How is the cause of a defect found in your company / from your experience in the press shop if the quality inspection at the end-of-line of the press reveals a surface defect (e.g. tearing) in the component?

	Manual process	Computer-aided	Automated	No evaluation possible
Type of defect detection	○	○	○	○

Does a part-related, clear labelling of blanks take place in your company / from your experience in the production of body shell parts?

- ◯ No evaluation possible
- ◯ No
- ◯ Yes, with the following method [ ]

Is blank-specific information (e.g. continuous sheet thickness, lubrication film measurement, etc.) currently used in the press shop in your company / from your experience to adjust the system parameters of the forming process?

- ◯ No evaluation possible
- ◯ No
- ◯ Yes

In which level of detail is a traceability of material parameters (e.g. sheet thickness, lubricant quantity etc.) in your company / from your experience currently possible?

- ◯ Coil related
- ◯ Batch related
- ◯ Blank / part related
- ◯ No allocation possible

Does your company/from your experience in the production of body shell parts carry out a part-related, clear labelling of the finished parts?

- ◯ No evaluation possible
- ◯ No
- ◯ Yes, with the following method [ ]

How do you evaluate the benefit potential of an automated data allocation of the machine parameters with the quality characteristics of the finished parts in the press shop in your company / from your experience?

	Low	High	No evaluation possible
Potential	◯	◯	◯

Unter Track & Trace wird die Technologie verstanden, welche Objekte entlang des Fertigungsprozesse erkennen und lokalisieren kann. Dabei ermöglicht die eindeutige Objektidentifikation eine Rückvervolgbarkeit der Bauteilhistrorie, sowie die Zurodnung eines spezifischen Datensatzes (Materialeigenschaften, Anlageneinstell- und Prozessparameter) zu jedem Bauteil.

By the use of Track & Trace technologies at blank / finished part level, would it be possible in the press shop in your company / from your experience to...

	Not applicable	Applicable	No evaluation possible
...increase transparency in the production flow?	◯	◯	◯
…reduce costs?	◯	◯	◯
...implement the component-specific transfer of manufacturing data?	◯	◯	◯
...automate the control efforts of the system control in the manufacturing process?	◯	◯	◯
...implement a blank specific press control?	◯	◯	◯
...reduce unscheduled downtime?	◯	◯	◯
…decrease the part-per-million defect rate?	◯	◯	◯

Which of the following statements is the biggest challenge when implementing a blank/part related Track & Trace system? Please arrange the statements in order.

Rating:
1- biggest to 4 - least challenge

- Upgrade of older production systems
- Labelling of blanks
- Labelling of finished parts
- Missing technologies for identification of blanks / finished components

» **Umleitung auf Schlussseite von Umfrage Online** (ändern)
