# Supplementary material for: Digital press shops: Data of an online survey among press shop experts
Source: Data Brief. 2021 Feb 13;35:106880. doi: 10.1016/j.dib.2021.106880 (PMC7907777; doi:10.1016/j.dib.2021.106880)
Supplement: Supplementary file 4 [file mmc4.pdf]

Fertigungsautomatisierung und Datenvernetzung im automobilen Presswerk

Unternehmens- und Teilnehmerinformationen

Digitale Technologie bieten neue Möglichkeiten für eine intelligente Produktion von Blechbauteilen. Insbesondere im Bereich der Fertigungsautomatisierung können durch vernetzte Produktionsprozesse neue Effizienz Potentiale gehoben werden.

Ziel dieser Umfrage ist es,
- den Ist-Zustand der einzelnen Fertigungsbereiche des automobilen Presswerks anhand ihrer Prozessautomatisierung zu charakterisieren,
- den Daten- / Informationsfluss von Materialeigenschaften und Fertigungsparametern im Presswerk zu beschreiben,
- die Schwerpunkte zur Umsetzung einer automatisierten geschlossenen Informationskette im Presswerk zu identifizieren.

Welcher Branche gehört Ihr Unternehmen an?

○ Automobil

○ Maschinen- und Anlagenbau

○ Software und IT

○ Logistik

○ Sonstige [ ]

Welche Beschreibung trifft Ihre Tätigkeit am besten?

○ Geschäftsführer

○ Manager

○ Fertigungsplaner

○ Entwicklungsingenieur

○ Qualitätsingenieur

○ Werker

○ Sonstige [ ]

Auf welcher Stufe in der automobilen Produktionskette steht Ihr Unternehmen?

- OEM
- Zulieferer
- Betriebsmittellieferant
- Sonstige ____________

Welche Größe hat Ihr Unternehmen?

- Kleinstunternehmen (< 10 Mitarbeiter)
- Kleines Unternehmen (10 bis 50 Mitarbeiter)
- Mittelgroßes Unternehmen (50 bis 250 Mitarbeiter)
- Großes Unternehmen / Konzern (> 250 Mitarbeiter)

Presswerk - Produktion

Welche Art von Pressen kommen in Ihrem Betrieb / aus Ihrer Erfahrung in der Fertigung von Karosserie Außenhautteilen überwiegend zur Anwendung?

- Weggebundene Pressen (bspw. Mechanische Hebel-, Kurbel-, Keil- und Exzenterpressen)
- Kraftgebundene Pressen (bspw. Hydraulikpressen)
- Keine Beurteilung möglich

Welche Angabe des Alters trifft auf die Pressenanlagen für Karosseriebauteile in Ihrem Betrieb / aus Ihrer Erfahrung im Presswerk am besten zu?

- < 3 Jahre
- 3 - 5 Jahre
- 6 - 15 Jahre
- > 15 Jahre

In welcher Form wird das Rohmaterial (Stahl / Aluminium) zur Fertigung von Karosserie Außenhautteilen in Presswerken in Ihrem Betrieb / aus Ihrer Erfahrung angeliefert?

- ◯ Coils
- ◯ Platinen
- ◯ Coils und Platinen
- ◯ Keine Beurteilung möglich

Die folgenden Fragen dienen der Charakterisierung der Automatisierungsausprägungen in den verschiedenen Fertigungsbereichen in Presswerken. Hierzu wurden 4 Stufen der Automatisierung definiert.

Händischer Prozess - Manuelle Umsetzung der Tätigkeit durch einen Werker (Bsp. Händisches Abstapeln von Bauteilen)

Maschinelle Unterstützung - Der Werker wird bei der Tätigkeit durch eine Maschine unterstützt. Die Maschine übernimmt dabei die Arbeitsbewegung, allerdings obliegen die Steuerung und Führung weiterhin dem Werker. (Bsp. Manuelle Steuerung des Hallenkrans zum Werkzeugwechsel)

Teilautomatisierung - Elektronische Steuerungen übernehmen sowohl die Arbeitsbewegung, als auch die Steuerung der Produktionsmaschinen. (Bsp. Manuelle Presswerkzeug-Auswahl, Hallenkran fährt automatisiert zur Werkzeugposition, Manuelle An- / Abkoppelung des Presswerkzeuges, automatisierte Positionierung am Zielort)

Vollautomatisierung - Die Automatisierungstechnik übernimmt die Aufgabe der Steuerung und Überwachung der technischen Prozesse vollumfänglich. (Bsp. Manuelle Presswerkzeug-Auswahl, Hallenkran fährt automatisiert zur Werkzeugposition, automatisierte An- / Abkoppelung des Presswerkzeuges, automatisierte Positionierung am Zielort)

Welcher der Automatisierungsbegriffe beschreibt für Sie die Bestandteile des Bereiches "Materialanlieferung" im automobilen Presswerk in Ihrem Betrieb / aus Ihrer Erfahrung am besten?

Der Bereich "Materialanlieferung" umfasst die Anlieferung der Materialen am Wareneingang, die Wareneingangsprüfung, die Zwischenlagerung, und die Intralogistik zur Materialbereitstellung zum nächsten Fertigungsbereich.

	Händischer Prozess	Maschinelle Umsetzung	Teilautomatisierung	Vollautomatisierung
Intralogistik Coil / Platinen	◯	◯	◯	◯
Materialeingangsprüfung	◯	◯	◯	◯

Welcher der Automatisierungsbegriffe beschreibt für Sie die Bestandteile des Bereiches "Bandschneidanlage" im automobilen Presswerk in Ihrem Betrieb / aus Ihrer Erfahrung am besten?

Der Bereich "Bandschneidanlage" erstreckt sich von der Materialbestückung der Anlage bis zur Intralogistik der Ladungsträger zur Zwischenlagerung oder zum nächsten Fertigungsbereich.

	Händischer Prozess	Maschinelle Umsetzung	Teilautomatisierung	Vollautomatisierung
Materialrüstvorgänge (Coil)	◯	◯	◯	◯
Schneidwerkzeug-Rüstvorgänge	◯	◯	◯	◯
Änderung der Anlagenparameter bei einer Qualitätsstörung	◯	◯	◯	◯
Abstapelung der Platinen	◯	◯	◯	◯
Intralogistik Platinen Ladungsträger	◯	◯	◯	◯

Welcher der Automatisierungsbegriffe beschreibt für Sie die Bestandteile des Bereiches "Pressenanlage" im automobilen Presswerk in Ihrem Betrieb / aus Ihrer Erfahrung am besten?

Der Bereich "Pressenanlage" erstreckt sich von der Materialbestückung der Anlage bis zur Intralogistik der Ladungsträger zum Fertigteillager.

	Händischer Prozess	Maschinelle Umsetzung	Teilautomatisierung	Vollautomatisierung
Intralogistik Platinen Ladungsträger	◯	◯	◯	◯
Platinen Rüstvorgänge	◯	◯	◯	◯
Werkzeug Rüstvorgänge	◯	◯	◯	◯
Änderung der Pressenparameter bei einer Qualitätsstörung	◯	◯	◯	◯
End-of-line Prüfung der Fertigbauteile	◯	◯	◯	◯
Abstapelung der Fertigbauteile	◯	◯	◯	◯
Intralogistik Fertigteil Ladungsträger	◯	◯	◯	◯

Welcher der Automatisierungsbegriffe beschreibt für Sie die Bestandteile des Bereiches "Fertigteillager" im automobilen Presswerk in Ihrem Betrieb / aus Ihrer Erfahrung am besten?

Der Bereich "Fertigteillager" umfasst die Intralogistik der Fertigteil-Ladungsträger, sowie die Abwicklung des Warenausgangs.

	Händischer Prozess	Maschinelle Umsetzung	Teilautomatisierung	Vollautomatisierung
Intralogistik Fertigteile Ladungsträger	◯	◯	◯	◯

Erfolgt im Presswerk in Ihrem Betrieb / aus Ihrer Erfahrung zwischen dem Fertigungsbereich "Pressenanlage" und dem vorgelagerten Fertigungsbereichs "Bandschneidanlage" ein automatisierter Datenaustausch zwischen den Anlagensystemen?

◯ Keine Beurteilung möglich

◯ Nein

◯ Ja

Wie bewerten Sie das Nutzenpotential einer Erhöhung des Automatisierungsgrades in den verschiedenen Bereichen der Prozesskette im Presswerk in Ihrem Betrieb / aus Ihrer Erfahrung?

	Gering	Hoch	Keine Beurteilung möglich
Materialanlieferung Coil / Platinen	◯	◯	◯
Bandschneidanlage	◯	◯	◯
Pressenanlage	◯	◯	◯
Pressenwerkzeug / Tooling Wechsel	◯	◯	◯
Pressenwerkzeug Instandhaltung	◯	◯	◯
Pressenanlagen Instandhaltung	◯	◯	◯
Lagerbereiche	◯	◯	◯
Intralogistik Ladungsträger Fertigteile	◯	◯	◯
Qualitätsregelkreise	◯	◯	◯
Daten- und Informationsfluss	◯	◯	◯

Presswerk - Daten- und Informationsflüsse im Presswerk

Auf welche Art erfolgt bei einer Prozessstörung (Bsp. Reißer an Fertigteil) an der Pressenanlage die Informationsweitergabe zwischen der End-of-line Prüfung und dem Anlagenführer in Ihrem Betrieb / aus Ihrer Erfahrung?

	Manuell (Werker erkennt manuell Prozessstörung, informiert Anlagenführer)	Technische Unterstützung (Anlagensystem erkennt mögliche Prozessstörung, Werker verifiziert)	Automatisiert (Anlagensystem erkennt und verifiziert die Prozesstörung)	Keine Beurteilung möglich
Art der Informationsweitergabe	◯	◯	◯	◯

Erfolgt im Presswerk in Ihrem Betrieb / aus Ihrer Erfahrung eine zentrale Datenspeicherung der Materialeigenschaften und Fertigungsparameter, sodass verschiedene Fertigungsbereiche der Produktion darauf zugreifen können?

- ◯ Keine Beurteilung möglich
- ◯ Nein
- ◯ Ja auf dem Anlagenspeicher
- ◯ Ja auf einem lokalen Datenserver
- ◯ Ja in einer Datencloud

Wie beurteilen Sie die aktuelle Dauer der Fehler-Ursachen-Findung in Ihrem Betrieb / aus Ihrer Erfahrung im Presswerk, wenn in der Qualitätsprüfung an der End-of-line der Presse ein Oberflächenfehler im Bauteil festgestellt wird?

	Gering	Hoch	Keine Beurteilung möglich
Dauer der Fehler-Ursachen-Findung	◯	◯	◯

Auf welche Art erfolgt die Fehler-Ursachen-Findung in Ihrem Betrieb / aus Ihrer Erfahrung im Presswerk, wenn in der Qualitätsprüfung an der End-of-line der Presse ein Oberflächenfehler (Bsp. Reißer) im Bauteil festgestellt wird?

	Manuell (Ursachensuche durch Anlagenführer)	Technische Unterstützung (Anlagensystem erkennt eine mögliche Ursache, Anlagenführer verifiziert)	Automatisiert (Anlagensystem erkennt und verifiziert die Ursache)	Keine Beurteilung möglich
Art der Informationsweitergabe	◯	◯	◯	◯

Erfolgt in Ihrem Betrieb / aus Ihrer Erfahrung in der Fertigung von Karosserie Außenhautteilen eine bauteilbezogene, eindeutige Kennzeichnung von Platinen?

◯ Keine Beurteilung möglich

◯ Nein

◯ Ja, mit Hilfe der folgenden Kennzeichnungsmethode

Werden aktuell im Presswerk in Ihrem Betrieb / aus Ihrer Erfahrung platinenspezifische Informationen (Bsp. kontinuierliche Blechdicke, Schmierfilmmessung usw.) zur Einstellung der Anlagenparameter des Umformprozesses genutzt?

◯ Keine Beurteilung möglich

◯ Nein

◯ Ja

In welchem Detaillierungsgrad ist eine Rückverfolgbarkeit von Materialeigenschaften (Bsp. Blechdicke, Schmiermittelmenge usw.) in Ihrem Betrieb / aus Ihrer Erfahrung aktuell möglich?

◯ Coil bezogen

◯ Chargen bezogen

◯ Platinen / Bauteil bezogen

◯ Keine Zuteilung möglich

Erfolgt in Ihrem Betrieb / aus Ihrer Erfahrung in der Fertigung von Karosserie Außenhautteilen eine bauteilbezogene, eindeutige Kennzeichnung der Fertigbauteile?

- ◯ Keine Beurteilung möglich
- ◯ Nein
- ◯ Ja, mit Hilfe der folgenden Kennzeichnungsmethode [ ]

Wie bewerten Sie das Nutzenpotential einer automatisierten Datenzuordnung der Anlagenparameter mit den Qualitätseigenschaften der Fertigbauteile im Presswerk in Ihrem Betrieb / aus Ihrer Erfahrung?

	Gering	Hoch	Keine Beurteilung möglich
Nutzenpotential	◯	◯	◯

Unter Track & Trace wird die Technologie verstanden, welche Objekte entlang des Fertigungsprozesse erkennen und lokalisieren kann. Dabei ermöglicht die eindeutige Objektidentifikation eine Rückvervolgbarkeit der Bauteilhistrorie, sowie die Zurodnung eines spezifischen Datensatzes (Materialeigenschaften, Anlageneinstell- und Prozessparameter) zu jedem Bauteil.

Durch den Einsatz von Track & Trace Technologien auf Platinen- / Fertigbauteilebene wäre es im Presswerk in Ihrem Betrieb / aus Ihrer Erfahrung möglich,

	Nicht zutreffend	Zutreffend	Keine Beurteilung möglich
...die Transparenz im Fertigungsfluss zu erhöhen.	○	○	○
...Kosteneinsparungen zu erzielen.	○	○	○
...die bauteilspezifische Weitergabe von Fertigungsdaten zu implementieren.	○	○	○
...die Kontrollaufwände der Anlagensteuerung im Fertiungsprozess zu automatisieren.	○	○	○
...eine platinenspezifische Pressensteuerung umzusetzen.	○	○	○
...ungeplante Störzeiten zu reduzieren.	○	○	○
...die Part-per-Million Fehlerrate zu reduzieren.	○	○	○

Welche der folgenden Aussagen stellt die größte Herausforderung bei der Implementierung eines Platinen- / Bauteilbezogenen Track & Trace Systems dar? Ordnen Sie bitte die Aussagen Ihrer Reihenfolge nach an.

Bewertung:
1- größte bis 4 - niedrigste Herausforderung

- Nachrüstbarkeit älterer Produktionsanlagen
- Markierung der Platinen
- Markierung der Fertigbauteile
- Fehlende Technologien zur Identifikation von Platinen / Fertigbauteilen

» Umleitung auf Schlussseite von Umfrage Online
